# Supplementary material for: Rapid subduction initiation and magmatism in the Western Pacific driven by internal vertical forces
Source: Nat Commun. 2020 Apr 20;11:1874. doi: 10.1038/s41467-020-15737-4 (PMC7170853; doi:10.1038/s41467-020-15737-4)
Supplement: Supplementary file 1 — Supplementary Information [file 41467_2020_15737_MOESM1_ESM.pdf]

Supplementary Information

Rapid subduction initiation and magmatism in the Western Pacific driven by internal vertical forces

B. Maunder[1], J. Prytulak[2], S. Goes[1] & M. Reagan[3]

1. Imperial College London, South Kensington, London, UK

2. Durham University, Durham, UK

3. University of Iowa, Iowa City, USA

Email: b.maunder@imperial.ac.uk

Symbol	Description	Value	Units
g	Acceleration due to gravity	9.8	ms^{-2}
ρ_m	Mantle density	3300	kgm^{-3}
α	Thermal expansivity	$3x10^{-5}$	K^{-1}
T_m	Asthenospheric temperature	1573	K
T_s	Surface temperature	273	K
κ	Thermal diffusivity	$1x10^{-6}$	m^2s^{-1}
t_{paci}	Age of Pacific plate	50	Myr
t_{phil}	Age of Philippine plate	5	Myr
R	Gas constant	8.31	$JK^{-1}mol^{-1}$
A_{df}	Rheological prefactor for diffusion creep	$3x10^{-11}$	$Pa^{-ndf}s^{-1}$
A_{ds}	Rheological prefactor for dislocation creep	$2.76x10^{-17}$	$Pa^{-nds}s^{-1}$
A_p	Rheological prefactor for Peierl's creep	$1x10^{-192}$	$Pa^{-np}s^{-1}$
n_{df}	Stress exponent for diffusion creep	1	-
n_{ds}	Stress exponent for dislocation creep	3.48	-
n_p	Stress exponent for Peierl's creep	24	-
E_{df}	Activation energy for diffusion creep	$3x10^5$	$Jmol^{-1}$
E_{ds}	Activation energy for dislocation creep	$4.5x10^5$	$Jmol^{-1}$
E_p	Activation energy for Peierl's creep	$3.94x10^5$	$Jmol^{-1}$
V_{df}	Activation volume for diffusion creep	$4x10^{-6}$	$m^3 mol^{-1}$
V_{ds}	Activation volume for dislocation creep	$1.4x10^{-5}$	$m^3 mol^{-1}$
V_p	Activation volume for Peierl's creep	$1x10^{-5}$	$m^3 mol^{-1}$
C	Cohesion (surface yield strength)	$1x10^7$	Pa
τ_{max}	Maximum yield strength	$8x10^8$	Pa
μ_0	Friction coefficient of undamaged material	0.6	-
μ_f	Friction coefficient of maximally damaged material	0.02	-
ε_f	Strain at which material is considered maximally damaged	0.8	s^{-1}
η_{min}	Minimum viscosity	$1x10^{19}$	Pas
η_{max}	Maximum viscosity	$1x10^{24}$	Pas
w_{fz}	Width of initial damaged fracture zone	10	km
d_{fz}	Depth of initial damaged fracture zone	40	km
d_{sed}	Thickness of sediment layer	1	km
d_{crust}	Thickness of crust	7	km
F_{pull}	Additional slab pull force applied to Pacific plate	9	TNm^{-1}
C_{H2O}	Concentration of water in mantle above dehydrating slab	0.05	Bulk wt%

Supplementary Table 1. All parameters that define the numerical model and the values given to them for this study.
